# An association between unrecognized gastroesophageal reflux disease and excessive daytime sleepiness in Taiwanese subjects suspected to have liver disease: a pilot study

**DOI:** 10.1186/1471-230X-11-55

**Published:** 2011-05-18

**Authors:** Jing-Hong Hu, Shih-Wei Lin, Yung-Yu Hsieh, Ning-Hung Chen

**Affiliations:** 1 Department of Gastroenterology and Hepatology, Chiayi Chang-Gung Memorial Hospital, No.6, W. Sec., Jiapu Rd., Puzi City, Chiayi County 613, Taiwan; 2 Department of Thoracic Medicine, Sleep Center, Chang-Gung Memorial Hospital, TaoYuan Chang-Gung Institute of Technology, Chang-Gung University, No.123, Dinghu Rd., Guishan Township, Taoyuan County 333, Taiwan

## Abstract

**Background:**

In traditional Chinese culture, liver disease is believed to underlie excessive daytime sleepiness (EDS). Consequently, Chinese patients with complaints of EDS and physicians who treat them suspect that a liver abnormality is present. If liver disease is ruled out, these patients are often discharged without treatment. Gastroesophageal reflux disease (GERD) is a common disorder also associated with EDS. This pilot study was undertaken to determine the prevalence of GERD among Taiwanese patients with complaints of EDS suspected to be related to liver disease but in whom no evidence for the latter was found.

**Methods:**

From July 2009 to December 2009, 121 outpatients who presented to or were referred to the Department of Gastroenterology and Hepatology of the Chiayi Gung Memorial Hospital for evaluation of a complaint of EDS thought to be due to liver disease were examined. Demographic data were collected, and physical examinations and liver function tests were performed. Forty-eight patients had liver disease and were excluded. The Chinese Epworth Sleepiness Scale questionnaire (Chinese ESS) and the Chinese Gastroesophageal Reflux Disease Questionnaire (CGERDQ) were then administered to 73 included patients.

**Results:**

More than half (56.2%) of the included patients were found to suffer from GERD. Patients with symptoms of GERD had higher mean CGERDQ scores than patients without symptoms of the disorder (18.88 ± 5.49 and 5.56 ± 3.57, respectively; *P *< 0.001). Patients with symptoms of GERD also had higher mean Chinese ESS scores than patients without symptoms (8.80 ± 5.49 and 3.13 ± 3.50, respectively; *P *< 0.001). Chinese ESS scores indicative of EDS were observed in 48.8% of patients with symptoms of GERD and in 3.1% of those without symptoms (*P *< 0.001). Differences between the two groups retained their significance after controlling for potential confounders.

**Conclusions:**

A significant percentage of Taiwanese patients who complained of EDS and were admitted to our Hepatology/Gastroenterology Department due to a suspicion of liver disease actually had symptoms of GERD. Further studies are needed to ascertain whether treatment of GERD will effectively resolve EDS in these patients.

## Background

Gastroesophageal reflux disease (GERD) is a common disorder affecting approximately 10-38% of the adult population in Western countries and an estimated 17% of adults living in Xi'an, Shaanxi province in China [1-3]. Consequences of GERD include, but are not limited to, Barrett's esophagus and adenocarcinoma of the esophagus. In addition, GERD is associated with upper respiratory disease, adversely affects quality of life (QOL), and represents a significant economic consideration in terms of treatment costs [1].

Sleepiness, or somnolence, is a complex state influenced by factors such as quantity and quality of prior sleep, circadian time, medications, and environmental stimuli as well as by medical, neurological, emotional, and psychiatric conditions. When sleep is not desired, somnolence is unwanted. Continuous inadequate sleep negatively impacts cognitive function, physical performance, overall well-being, and QOL [4]. The pathologic or inappropriate occurrence of somnolence is clinically referred to as excessive daytime sleepiness (EDS) [5].

Sleep contributes to a reduction in normal anti-reflux mechanisms; rates of swallowing and salivation are decreased, pressure from the upper and lower esophageal sphincters is decreased, gastric emptying is delayed, and the "heartburn-signal" is depressed. The supine position of most sleepers is thought to facilitate gastroesophageal reflux, a major cause of disrupted sleep due to awakenings from heartburn, dyspepsia, acid brash, coughing, or choking [5,6]. Not unexpectedly, more than half of patients with chronic GERD report nocturnal symptoms [2-4,7-10]. The consequences of nocturnal GERD are thought to exceed those of daytime GERD in terms of increased risk of esophageal lesions and respiratory complications, poor QOL, inadequate sleep, EDS, poor work productivity, and increased costs. Like daytime GERD, nocturnal GERD can be managed with surgical therapies or proton pump inhibitors such that heartburn and regurgitation are reduced concurrently with an improvement in sleep.

In addition to GERD, EDS is associated with other co-morbidities including liver disease [11-17]. Daytime fatigue is frequently encountered in patients carrying hepatitis virus and in those with primary biliary cirrhosis, cirrhosis without hepatic encephalopathy, and nonalcoholic fatty liver disease. In traditional Chinese culture, liver disease is believed to underlie sleep disorders. Consequently, Chinese patients with complaints of daytime sleepiness and physicians who treat such patients suspect that a liver abnormality is present. Unfortunately, if liver disease is ruled out, these patients are often discharged without treatment or follow-up examinations.

The present pilot study was undertaken to determine the prevalence of GERD among patients who presented to or were referred to the Department of Gastroenterology and Hepatology with complaints of daytime sleepiness suspected to be related to an underlying liver disease but in whom no evidence for liver disease was obtained.

## Methods

### Patients

From July 2009 to December 2009, outpatients who either presented to or were referred to the Department of Gastroenterology and Hepatology of the Chiayi Gung Memorial Hospital for evaluation of a previously uninvestigated sleep disorder were included. In Taiwan, an official referral to a gastroenterology/hepatology department is not required; therefore patients in this study were admitted with or without a referral. Exclusion criteria were: previous diagnosis with any type of sleep disturbance; diagnosis of GERD; current treatment for GERD by a physician; and previous abnormal liver function test findings. Patients known to have chronic hepatitis B or C or to be carriers of hepatitis B or C were also excluded. Written informed consent was obtained from all patients, and this study was approved by the Ethical Committee for Human Clinical Trials of the Chiayi Gung Memorial Hospital, R.O.C. (Trial Number 98-2033C).

Demographic data were collected at baseline, including information regarding use of medications, alcohol, and tobacco. A physical examination was performed, and a blood sample was collected from each patient for measurements of hepatitis B serum antigen (HBsAg), anti-HCV Ab, aspartate aminotransferase (AST), and alanine aminotransferase (ALT). Patients with any abnormal measurements were then excluded. None of these patients had a disease history warranting additional tests (such as a complete blood count). Furthermore, additional tests were not permitted by the insurance policies of these patients.

### Questionnaires

The Chinese Epworth sleepiness scale questionnaire (Chinese ESS) was administered to all included patients by the authors. The Chinese ESS is based on the Epworth sleepiness scale for measurement of daytime sleepiness; the latter is considered to be the most discriminating test for measurement of EDS [18-21]. Designed for diagnosis of EDS in all Chinese subjects, the Chinese ESS has been validated for use in Taiwanese and Mandarin Chinese populations [22]. Specifically, the Chinese ESS measures the propensity for sleep under a variety of life-related situations [21]. The questionnaire consists of eight self-reported questions, with each receiving a score of 0 to 3. Total possible scores range from 0 (the least sleepy) to 24 (the most sleepy), with a total score of 5.9 ± 2.2 considered normal for this group of subjects [19,20]. A score of 10 is indicative of EDS and is considered the threshold for referral for sleep evaluation [21].

The Chinese Gastroesophageal Reflux Disease Questionnaire (CGERDQ) was also administered to all patients included in the study. This questionnaire, which is designed to identify individuals with symptoms of gastroesophageal reflux, is comprised of eight questions. Each question evaluates the frequency and severity of a symptom of GERD and is scored from 0-3, with a total score ≥12 serving to indicate the presence of GERD [23]. The questionnaire has been validated for use in Chinese subjects and has been shown to be capable of detecting changes in symptoms [23,24].

### Statistical analyses

Group comparisons for normally distributed continuous variables between subjects with and without GERD symptoms were performed using independent two-sample t tests. The Chi-square test or Fisher's exact test was used for comparisons of categorical variables. Normally distributed continuous data are presented as means ± standard deviation whereas categorical data are represented by numbers and percentage. Stepwise linear regression analysis was performed to identify the factors influencing the Chinese ESS. All statistical assessments were two-sided, with a *P *value of < 0.05 indicating statistical significance. Statistical analyses were performed using SPSS 15.0 statistics software (SPSS Inc., Chicago, IL, USA).

## Results

Between July 2009 and December 2009, 121 consecutive patients with a primary complaint of EDS were initially included. In every case, either the patient and/or the referring practitioner suspected that the complaint was related to liver disease. Of these, 48 patients were subsequently excluded because of the presence of chronic liver disease. Specifically, chronic HBV hepatitis was found in 12 patients (of which 5 also had fatty liver), HCV hepatitis was found in 13 patients (of which 6 also had fatty liver), HBV and HCV hepatitis were found to be present in 1 patient, and fatty liver only was found in the remaining 22 patients. Seventy-three consecutive patients without liver disease who presented to or were referred to the outpatient Hepatology and Gastroenterology Clinic with a primary complaint of EDS were then selected for this study. The baseline characteristics of these 73 subjects are presented in Table 1. This group included 34 (46.6%) males and 39 (53.4%) females ranging in age from 17 to 78 years (mean = 45.75 ± 17.82 years). Based on the results of the CGERDQ, GERD symptoms were present in 41 (56.2%) of the 73 subjects. Mean CGERDQ scores in the GERD and non-GERD groups were 18.88 ± 5.49 and 5.56 ± 3.57, respectively (*P *< 0.001). The GERD group also contained more patients with positive Chinese ESS scores (>10) as compared to those in the non-GERD group (48.8% *versus *3.1%, *P *< 0.001). The mean Chinese ESS score for subjects with GERD was higher than that for subjects without the disorder (8.80 ± 5.49 *versus *3.13 ± 3.50, *P *< 0.001). Most subjects included in this study did not use alcohol (71.2%), acid suppression (75.3%), or sedatives/hypnotics (95.9%). In addition, 69.9% and 91.8%, respectively, of subjects in this study did not smoke or snore. All subjects were found to be negative for HBsAg and Anti-HCV Ab, and AST and ALT values for all subjects were within the normal range.

**Table 1:** Baseline characteristics of 73 patients with or without gastroesophageal reflux disease as determined by the Chinese Gastroesophageal Reflux Disease Questionnaire

	Total (*n *= 73)	GERD Symptoms (*n *= 41)	No GERD Symptoms (*n *= 32)	*P *value
Age (years)^a^	45.75 ± 17.82	49.95 ± 18.33	45.50 ± 17.43	0.915
Gender, n (%)^b^				
Male	34 (46.6)	16 (39.0)	18 (56.3)	0.143
Female	39 (53.4)	25 (61.0)	14 (43.8)	
BMI (kg/m^2^)^a^	23.19 ± 3.60	22.95 ± 3.77	23.50 ± 3.41	0.522
Chinese ESS^b^				
≤10	52 (71.2)	21 (51.2)	31 (96.6)	<0.001
>10	21 (28.8)	20 (48.8)	1 (3.1)	
CGERDQ score^a^	13.04 ± 8.00	18.88 ± 5.07	5.56 ± 3.57	<0.001
Alcohol, *n *(%)^b^				
Yes	21 (28.8)	11 (26.8)	10 (31.3)	0.679
No	52 (71.2)	30 (73.2)	22 (68.8)	
Smoking, *n *(%)^b^				
Yes	22 (30.1)	12 (29.3)	10 (31.2)	0.855
No	51 (69.9)	29 (70.7)	22 (68.8)	
Acid suppression, *n *(%)^b^				
Yes	18 (24.7)	15 (36.6)	3 (9.4)	0.007
No	55 (75.3)	26 (63.4)	29 (90.6)	
Snoring, *n *(%)^c^				
Yes	6 (8.2)	6 (14.6)	0 (0.0)	0.032
No	67 (91.8)	35 (85.4)	32 (100.0)	
S/H use, *n *(%)^c^				
Yes	3 (4.1)	3 (7.3)	0 (0.0)	0.251
No	70 (95.9)	38 (92.7)	32 (100.0)	
AST (U/L)	22.10 ± 5.65	21.54 ± 5.88	22.81 ± 5.35	0.342
ALT (U/L)	22.03 ± 6.91	21.56 ± 7.16	22.63 ± 6.63	0.517

Findings are presented either as means ± standard deviation or as numbers (%).

*P *values were determined from ^a^independent two sample t tests, ^b^Chi-square tests, or ^c^Fisher's exact test.

Abbreviations: BMI, body mass index; Chinese ESS, Chinese Epworth Sleepiness Scale; CGERDQ, Chinese Gastroesophageal Reflux Disease Questionnaire; S/H, sedatives and/or hypnotics

Simple and multiple linear regression analyses were performed to identify factors influencing the Chinese ESS. Findings of these analyses are presented in Table 2. The only variable found to influence the Chinese ESS significantly was the CGERDQ score (*P *< 0.001). After controlling for the potential confounders of age and gender, the CGERDQ score remained significantly and positively associated with the Chinese ESS. The adjusted *R*^2 ^was 0.426, indicating that the statistical modeling accounted for approximately 43% of the variation in the Chinese ESS.

**Table 2:** Results of simple and multiple linear regression analyses to identify factors influencing the Chinese Epworth Sleepiness Scale

	Simple		Multiple	
	Coefficient (SE)	*P *value	Coefficient (SE)	*P *value
Age	0.004 (0.037)	0.921	-0.005 (0.082)	0.850
Gender	0.842 (1.292)	0.517	1.151 (0.983)	0.246
BMI	0.094 (0.180)	0.605		
CGERDQ score	0.454 (0.061)	<0.001	0.457 (0.061)	<0.001
Alcohol	-1.111 (1.422)	0.437		
Smoking	-2.273 (1.383)	0.105		
Acid suppression	1.204 (1.493)	0.423		
Snoring	2.017 (2.341)	0.392		
S/H use	1.410 (3.252)	0.666		
AST	0.027 (0.115)	0.813		
ALT	-0.023 (0.094)	0.806		

Abbreviations: BMI, body mass index; CGERDQ, Chinese Gastroesophageal Reflux Disease Questionnaire; S/H, sedatives and/or hypnotics

## Discussion and Conclusion

In this pilot study, 73 Taiwanese subjects with complaints of EDS but for whom no evidence of liver disease was obtained were examined for the presence of GERD; remarkably, more than half (56.2%) were found to suffer from this disorder. Mean CGERDQ scores for patients with symptoms of GERD (GERD group) were 3.4-fold higher than those for patients without symptoms of the disorder (non-GERD group). Patients in the GERD group were found to have significantly higher mean Chinese ESS scores than those in the non-GERD group. Additionally, more patients in the GERD group had Chinese ESS scores that exceeded 10. The differences in CGERDQ and Chinese ESS scores between the GERD and non-GERD groups retained their significance after controlling for the potential confounders of age and gender. While a cause and effect relationship remains to be established, these findings support an association between GERD and EDS in Taiwanese subjects without liver disease.

The mean ESS score for the entire cohort of patients examined in this study (6.32 ± 5.49) was comparable to that reported for the normal community (5.9 ± 2.2) [19,20] whereas mean scores for patients in the GERD and non-GERD groups were 8.80 ± 5.49 and 3.13 ± 3.50, respectively. It is unclear why the mean score for the GERD group fell below that considered to indicate EDS and why the mean score for the non-GERD group fell below that for the normal community. It is possible that some of the subjects in this study who claimed to have EDS did not in fact suffer from more daytime fatigue than do members of the local population. Additionally, some patients in this study may have not been overly concerned about their daytime sleepiness and therefore presented directly to a gastroenterologist in preference to a sleep center.

A relationship between GERD and daytime somnolence has been described in other studies. For example, Demeter *et al. *[4] reported that the severity of GERD as assessed by panendoscopy and characterized by the Savary-Miller classification correlated positively with the degree of daytime somnolence as assessed by the ESS. Specifically, mild somnolence was observed in 29% of GERD 0 patients, 39% of GERD I patients, and 46% of GERD II patients; however 77% of GERD III patients suffered from significant somnolence. Guda *et al. *[25], who evaluated 385 consecutive subjects at an outpatient clinic for sleep disorders, reported that those with a diagnosis of GERD had significantly higher ESS scores than those diagnosed without GERD. Furthermore, Chen *et al. *[26] reported that patients with nighttime heartburn complain of significantly greater subjective sleep impairment as determined by the Pittsburgh Sleep Quality Index as compared to those without nighttime heartburn. However, it should be noted that Suurna *et al. *[27] observed no differences between patients with and without nighttime heartburn in any objective sleep parameter during an overnight polysomnographic study.

In a recently published systematic review of the literature [10], the mean prevalence of nocturnal GERD was reported to be 54%. Farup *et al. *[3], who conducted a national random-sample telephone survey, reported that 71% of subjects with GERD experienced nocturnal heartburn. In a separate study [28] of 1,000 individuals who experienced heartburn at least once weekly, the prevalence of nocturnal heartburn was found to be 79%; three-quarters of those with nocturnal heartburn revealed that their heartburn episodes negatively impacted their sleep. In the present study, the prevalence of nocturnal as compared to daytime GERD was not directly determined. No patient included in this study complained of nocturnal heartburn but all were troubled by EDS such that the assistance of a gastroenterologist was sought. Based on inquiries conducted after completion of the questionnaires, it was apparent that all subjects in the study were likely to have experienced at least light nocturnal heartburn approximately once weekly. Unfortunately, no accepted questionnaire is currently available to define the degree of nocturnal heartburn, and patients in this study were not willing to submit to measurements of nighttime heartburn with instruments required for esophageal testing. Future studies focusing on consequences of nocturnal as opposed to daytime GERD are indicated; nocturnal symptoms are associated with significant impairment of health-related QOL and nocturnal acid reflux is associated with severe esophagitis, stricture, and esophageal adenocarcinoma [3,28].

As reviewed by Orr [10], consequences of reflux events include difficulty initiating sleep, arousals from sleep, daytime fatigue, and impaired work productivity. It therefore appears reasonable to consider sleep-related GERD a distinct clinical entity [10]. Fortunately, GERD can be effectively managed using such approaches as head of bed elevation, proton pump inhibitor therapy, H_2 _receptor antagonist therapy, and Nissen funoplication [5]. According to the ProGERD initiatives [2], the impairment of QOL by GERD can be significantly attenuated or normalized in as little as two weeks by treatment with esomeprazole. In the present study, significantly more patients in the GERD group as compared to the non-GERD group utilized acid suppression (15% as compared to 9.4%, *P *= 0.007). This finding further supports the hypothesis that GERD contributes to EDS in Taiwanese subjects.

The present study is unique in that subjects were included who complained of EDS rather than symptoms of GERD. Considering the negative impact of EDS, the high prevalence of GERD [1,2], and the clear association between GERD and fatigue [4,7], it would appear prudent for general practitioners to consider GERD as an underlying contributor to daytime somnolence in patients with normal liver function. Following a diagnosis of GERD, initiation of appropriate therapy and subsequent evaluation of that therapy would be indicated. In support of these recommendations, pantoprazole was found to provide more significant reductions in reflux symptoms and ESS scores than placebo after administration to patients with mild-moderate obstructive sleep-disordered breathing and GERD [27].

The presence of snoring and sleep apnea are relevant to an evaluation of EDS. In the present study, 14.6% of patients in the GERD group but 0% of patients in the non-GERD group reported snoring. According to patients in the GERD group, snoring was neither frequent nor severe. However, no tests were performed to determine either the accuracy of these assessments or the presence of obstructive sleep apnea in these subjects. Further research is warranted to determine the relative extents to which GERD and sleep apnea contribute to EDS in Taiwanese subjects and other populations.

The most important limitations of this study are the small sample size, the absence of confirmation of a diagnosis of GERD by endoscopy or other appropriate diagnostic test, and the lack of a control group. This pilot study was designed to determine the prevalence with which Taiwanese patients without liver disease but who complain of EDS exhibit symptoms of GERD. Furthermore, endoscopy is not routinely performed in hepatology clinics in Taiwan. Future randomized, controlled clinical studies that include a larger number of patients, an appropriate control group, and tests confirming the presence of GERD are clearly warranted. In addition, the present study did not evaluate QOL or social or psychological factors with the potential to contribute to EDS. Given the demonstrated negative impact of both GERD and daytime somnolence on QOL, such evaluations are also warranted.

EDS is a common complaint of patients with liver disease [12-17]. However, Chinese patients with complaints of EDS but subsequently found to have normal liver function are often left untreated. In these patients, EDS persists and the underlying cause is left undiagnosed. This small pilot study reveals that many patients with complaints of EDS and who were admitted to our Department of Hepatology and Gastroenterology due to a suspicion of liver disease actually had symptoms of GERD. Further studies are needed to ascertain whether appropriate treatment of GERD will effectively resolve EDS in these patients.

## Competing interests

The authors declare that they have no competing interests.

## Authors' contributions

JHH designed the study, prepared the protocol, and wrote the first draft of the paper. SWL revised the study design. YYH collected the data. NHC directed and supervised the performance of the study and the writing of the paper. All authors have approved the final draft of this paper.

## Pre-publication history

The pre-publication history for this paper can be accessed here:

http://www.biomedcentral.com/1471-230X/11/55/prepub
